# Focused extracorporeal shockwave therapy for youth sports-related apophyseal injuries: case series

**DOI:** 10.1186/s13018-023-04065-0

**Published:** 2023-08-22

**Authors:** Tarek Shafshak, Marwa Abdullah Amer

**Affiliations:** Department of Physical Medicine, Rheumatology and Rehabilitation, Faculty of Medicine, Alexandria University, Al-Khartoum Square, Alexandria, 21526 Egypt

**Keywords:** Extracorporeal shockwave therapy, Overuse injuries, Sports, Osgood-Schlatter disease

## Abstract

**Background:**

Apophyseal overuse injuries are self-limited with skeletal maturity; however, they may be a source of significant pain and time lost from training. There is a lack of consensus for its management with the current available treatment, which might lag behind the ongoing development of regenerative medicine. The current retrospective case study aimed to assess the potential effectiveness and short-term safety of extracorporeal shockwave therapy (ESWT) in apophyseal injuries.

**Methods:**

Data from 22 growing athletes [15 patients with Osgood-Schlatter disease and seven patients with Sever’s disease] who received ESWT at a sports medicine unit in a university hospital were reviewed. All patients received low energy (= 0.1 mJ/mm^2^) level-focused ESWT using electrohydraulic generation technology. The clinical focusing technique was used upon applying ESWT.

**Results:**

The number of sessions received to achieve full recovery ranged from 1 to 3 sessions. The time from treatment initiation to previous activity level was 2 weeks in 14 patients (63.3%), 4 weeks in seven patients (31.8%) and 11 weeks in one patient (4.5%). No adverse events were reported. No recurrence occurred up to 3 months after the last session.

**Conclusion:**

ESWT is a potentially safe and effective treatment for apophyseal injuries. It may facilitate an early return to sport activities.

## Background

Traction apophyseal injuries are the most frequent growth-related overuse injuries [1]. These injuries are still believed to be short-lived with self-resolving symptoms, although they could be a source of significant discomfort, pain and time lost from training in young athletes with possible recurrence for 12–18 months before complete resolution at skeletal maturity [2]. The dogma that closure of the growth plates with skeletal maturity will cause the symptoms to disappear has led to the commonly perceived innocuous nature of these injuries with no consensus for their management in young athletes [3]. Current treatment might include a ‘wait and see’ strategy or an extended period of rest during which multimodal conservative measures such as non-steroid anti-inflammatory medications, bracing, physical therapy and exercises might be beneficial [3]. This treatment could prove outdated with the ongoing development of regenerative orthopedic medicine.

Extracorporeal shockwave therapy (ESWT) is an emerging tool in regenerative musculoskeletal medicine with very promising results [4]. Currently, data on the role of ESWT in the treatment of apophysitis are scarce. The aim of this retrospective study is to demonstrate the effectiveness and short-term safety of focused ESWT in overuse apophyseal injuries.

## Materials and methods

Medical records of the Sports Medicine unit at the Physical Medicine and Rehabilitation Department of a university hospital from January 2011 to July 2019 were analyzed in a retrospective study. Patients diagnosed with apophysitis who received ESWT were included in this study. A total of 22 patients (21 boys and one girl), aged 11–15 years (mean ± SD = 13.3 ± 1.5; median = 14 years) diagnosed with apophysitis based on history, clinical presentation, and plain radiographs, were included in the study [5]. Fifteen patients (68.2%) had Osgood-Schlatter disease (OSD), and seven (31.8%) patients had Sever’s disease. The diagnosis of OSD was established based on clinical presentation (anterior knee pain and/or swelling where pain was exacerbated with physical activity such as running, jumping, and kneeling), tenderness over tibial tuberosity with or without enlargement and plain radiograph to confirm the diagnosis (fragmentation of the apophysis). Sever’s disease was diagnosed in patients with posterior heel pain (mainly activity-related pain), swelling and positive squeeze test where pain was induced by medial and lateral compression of the heel. Diagnosis was confirmed in all studied patients by plain radiograph that showed either bone fragmentation or sclerosis of the calcaneal apophysis. The duration of symptoms prior to ESWT ranged from 2 to 24 months (mean ± SD = 12.5 ± 8.87; median = 12 months). Twenty-one patients were competitive athletes, and one patient was a recreational soccer player.

All patients received focused (f-ESWT) using an electrohydraulic machine with advanced spark wave technology (ASWT) [OrthoWave 180 C^ASWT^]. Patients received 1–3 sessions at weekly intervals, low energy level [energy flux density (EFD) = 0.1 mJ/mm^2^], frequency = 4 Hz and 1100–2000 shocks/session, depending on patients’ tolerance. Applicator CE 50 (focused) was applied at the point of maximum tenderness recorded by palpation (clinical focusing technique) with a coupling layer of ultrasound gel. Shockwave therapy was focused only on the apophyses away from the growing epiphyses. The applicator was applied with an angle (directed downward) over the medial and lateral sides of the apophyses. The growing long bone epiphyses were away from the field of f-ESWT. In OSD, the applicator was placed at least 1–2 cm away from the epiphyseal plate of the growing knee (proximal tibia). In patients with Sever’s disease, f-ESWT was applied about 3 cm away from the fibular or tibial epiphysis over the medial and lateral surfaces of the posterior calcaneus (50% of shocks were given on each side) along the apophyseal zone. The sessions were given by the authors (physiatrists well-trained for ESWT ≥ 10 years). No anesthesia was used during ESWT application. All patients were instructed to stop their sport activities (avoid running or activity causing stress on the involved apophysis) for 2 weeks after each session and to use the appropriate brace [infrapatellar strap for OSD and heel rise for Sever’s disease]. Gradual return to sport activities was advised after complete pain relief with the following instructions: appropriate shoe modifications, stretching exercises should be performed prior to training, and training should be stopped immediately once pain develops. Assessment of pain improvement and return to sport and recreational activities was performed 1 week after each session and up to 3 months after the last session.

The following data were recorded: total number of sessions received, number of shocks/session, total number of shocks received through the entire course, total energy dose (TED) delivered per treatment (calculated by multiplying EFD by the total number of shocks) [6], total energy EFD (calculated as the product of the number of treatment sessions, the number of impulses per treatment session and the EFD of the impulses) [7], percent of pain improvement after each session [using a scale from zero (= no improvement) up to 100% (= complete relief)], return to play time, any adverse events and recurrence.

The study was approved by the ethical committee, and confidentiality of records was considered.

Data were analyzed using SPSS (IBM Corp. Released 2020. Statistics for Windows, Version 27.0. IBM Corp. Armonk, NY). The distributions of quantitative variables were tested for normality using the Kolmogorov–Smirnov test. Normally distributed data are described using the mean and standard deviation, while nonnormally distributed data are described using the median and range. Frequency (percent) was used for qualitative data.

## Results

The characteristics of ESWT sessions are given in Table 1. After the first session, the percentage of pain relief ranged from 50 to 100% (median = 100%) in 21 patients, and only one patient developed flare. Complete pain relief was achieved in 14 patients (63.3%) after a single ESWT; (Table 2). In the remaining patients, pain relief was complete 1–2 weeks after the second session in seven patients (31.8%) and was 70% in only one patient who achieved complete relief later after the third session.

**Table 1:** Characteristics of ESWT sessions among the studied patients, *n* = 22

	OSD (*n* = 15)	Sever’s disease (*n* = 7)
*Total number of sessions received*		
Median (min–max)	1(1–3)	1(1–2)
*Number of shocks/session*		
Mean ± SD	1506.6 ± 173.1	1300 ± 177
*TED/session (mJ/mm*^*2*^*)*		
Median (min–max)	150 (120–200)	120 (110–150)
*Total EFD (mJ/mm*^*2*^*)*		
Median (min–max)	150 (150–360)	120 (110–300)

*OSD* Osgood-Schlatter disease, *TED/session* Total energy dose/session, *Total EFD* Total energy flux density

**Table 2:** Percent of pain relief after the first session in OSD and Sever’s disease

	100% pain relief, *n* (%)		90% pain relief, *n* (%)		50% pain relief, *n* (%)		No improvement, *n* (%)	
	OSD	Sever’s	OSD	Sever’s	OSD	Sever’s	OSD	Sever’s
	(*n* = 15)	(*n* = 7)	(*n* = 15)	(*n* = 7)	(*n* = 15)	(*n* = 7)	(*n* = 15)	(*n* = 7)
7–10 days	8 (53.3)	4 (57.1)	3 (20)			2 (28.6)		
14–< 21 days	1 (6.7)	1 (14.2)			1 (6.6)			
21–< 28 days					1 (6.6)		1 (6.7)	
Total, *n* (%)	**9 (60)**	**5 (71.4)**	**3 (20)**		**2 (13.3)**	**2 (28.6)**	**1 (6.7)**	

*OSD* Osgood-Schlatter disease

**Fig. 1:**
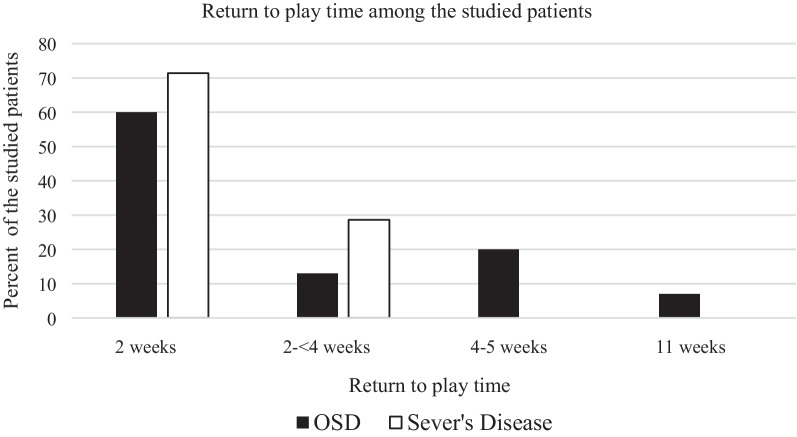
Time interval from treatment initiation until full recovery among the studied patients.  (Groups: 2 weeks/ >2-<4 weeks/ 4-5 weeks/ 11 weeks)

Among the studied patients, return to play time ranged from 2 to 11 weeks (median = 2 weeks) after ESWT sessions; (Fig. 1). This was maintained for 3 months with no recurrence. No adverse events were reported in any patient. There were no skeletal deformities for 3 months of follow-up after the last session.

## Discussion

The current study showed that focused ESWT is a safe noninvasive effective treatment for overuse apophyseal injuries. Only 1–2 sessions with adequate relative rest (limiting sport-specific repetitive movements) could be sufficient for healing, symptom resolution and early return to sport activities.

Until recently, ESWT over growth zones was strictly contraindicated based on the conclusion of a historical study in animals [8]. However, further studies showed no negative histological changes, and the authors concluded that human application of ESWT on apophyses in the growing age would be harmless and therapeutically effective [9, 10]. Consequently, there was a paradigm shift in ESWT in apophysitis from “prohibition” to “an exceptional indication/expert indication” in 2008 [11]. With the first clinical case series that reported both safety and efficacy of radial pressure therapy (RPT) in OSD and Sever's disease [12, 13], ESWT has been considered among “common empirically tested clinical uses” in treating apophysitis [14].

The current study revealed that more than two thirds of the patients may have complete relief after only one single session. While this could be explained by the fact that children heal quicker and faster than adults [15–17], the total number of sessions received in this study (1–3) is lower than what was reported previously. Lohrer et al. [12] reported 3–7 sessions (1500–2000 impulses) for patients with OSD, and Lohrer et al. [13] reported 2–5 sessions (1200–2000 impulse) for patients with Sever’s disease. The TED utilized in this study for OSD was slightly higher than that reported by Lohrer et al. [12] (120–200 mJ/mm^2^ vs. 90–180 mJ/mm^2^, respectively). These differences could be attributed to the use of different generation technologies. In this study, an electrohydraulic generation technology with ASWT was used, while other studies used pneumatically generated radial pressure waves. It is noteworthy that in the literature, RPT might have been established as ESWT, but this is not correct from a physical point of view. ESWT and RPT are different treatments independently with different biological effects [18, 19].

Scheduled rest away from training and competition for 2–3 months is considered the main treatment and prevention of overuse injuries in youth [20]. In this study, there was rapid improvement following ESWT without recurrence up to 3 months, although there were patients who complained for 2 years. Most of the studied patients resumed their sport activity 2–4 weeks after treatment. Only one patient had a prolonged recovery time (11 weeks) to reach the previous activity level. This patient was practicing soccer as a recreational activity. He had prolonged intervals between the three sessions that he received as he was living away from the hospital offering ESWT; therefore, he might have such a prolonged recovery time.

The current study demonstrated the potential short-term benefits of ESWT in apophyseal injuries. While Lohrer et al. [12, 13] reported the safety and efficacy of RPT in OSD and Sever’s disease several years after treatment (3.4–6.7 years and 1–8 years, respectively), this does not rule out the fact that only the spontaneous course of the disease was responsible for the improvement reported in their studies.

Repetitive traction forces placed over these growing apophyses have been proposed as the main pathophysiology underlying apophyseal overuse injuries. These traction forces result in chronic irritation, inflammation, and microavulsions at the bone-cartilage junction [5]. Without sufficient recovery time, these repetitive microinjuries overwhelm the normal reparative processes and develop into apophysitis. All these pathological processes are considered the perfect scenario for ESWT.

ESWT can enhance pain relief and long-term analgesia, lasting between several months and years, following a couple of ESWT sessions. Various mechanisms have been proposed, including: (i) modulation of substance P release, (ii) reduction in pain mediators such as calcitonin-gene-related peptide and (iii) hyperstimulation analgesia, which alleviates pain as a result of moderate-to-intense sensory input that is usually applied at the site of greatest discomfort [21].

ESWT can promote healing, and tissue regeneration via “mechanotransduction” [22]. The mechanical load applied to the cytoskeleton triggers angiogenic and healing responses at the cellular and molecular levels with increased protein biosynthesis and release of different growth factors, such as transforming growth factor ß1 (TGF-ß1), vascular endothelial growth factor (VEGF) and nitric oxide (NO) [23]. Moreover, it has been reported that low-energy ESWT stimulates a polarity shift in the macrophage phenotype from M1 (proinflammatory) to M2 (anti-inflammatory) [24]. This is particularly valuable as M2 macrophages are directly involved in regenerative processes after injury [25].

The treatment protocol used in this study was tailored to each patient as there were no previous protocols recommended for apophyseal injuries using focused ESWT. It was observed that this “individualized protocol” allowed the progress of treatment with patient-guided feedback.

The current study has limitations. First, there was no control group to compare the efficacy of ESWT to other treatments or placebo. Second, the sample size was small. Third, there were no data available for specific functional evaluation assessment tools. Last, the follow-up duration was only 3 months.

## Conclusions

Focused ESWT is a noninvasive technique that can be repeated with good tolerability by youth athletes while also lacking side effects. It may shorten healing times and enhance an early return to play hence providing an alternative to the usual “wait and see” strategy. While this study demonstrated potential short-term efficacy and safety of ESWT in apophyseal injuries, it is recommended to conduct a prospective controlled trial with long-term follow-up (at least 1 year) to clarify the long-term efficacy and further investigate any adverse events of f-ESWT on the growing epiphyses.

## Data Availability

The datasets used and/or analyzed during the current study are available from the corresponding author on reasonable request.

## Abbreviations

ESWT: Extracorporeal shockwave therapy
f-ESWT: Focused extracorporeal shockwave therapy
ASWT: Advanced spark wave technology
EFD: Energy flux density
OSD: Osgood-Schlatter disease
TED: Total energy dose
TGF-ß1: Transforming growth factor ß1
VEGF: Vascular endothelial growth factor
NO: Nitric oxide

## References

[1] Naaktgeboren K, Dorgo S, Boyle JB (2017). Growth plate injuries in children in sports: a review of sever’s disease. Strength Cond J.

[2] Caine D, DiFiori J, Maffulli N (2006). Physeal injuries in children’s and youth sports: reasons for concern?. Br J Sports Med.

[3] Holden S, Rathleff MS (2020). Separating the myths from facts: time to take another look at osgoodschlatter “disease”. Br J Sports Med.

[4] Moya D, Ramón S, Schaden W, Wang CJ, Guiloff L, Cheng JH (2018). The role of extracorporeal shockwave treatment in musculoskeletal disorders. J Bone Jt Surg Am.

[5] Hoang QB, Mortazavi M (2012). Pediatric overuse injuries in sports. Adv Pediatr.

[6] Tam KF, Cheung WH, Lee KM, Qin L, Leung KS (2005). Delayed stimulatory effect of low-intensity shockwaves on human periosteal cells. Clin Orthop Relat Res.

[7] Schmitz C, Császár NBM, Milz S, Schieker M, Maffulli N, Rompe JD, Furia JP (2015). Efficacy and safety of extracorporeal shock wave therapy for orthopedic conditions: a systematic review on studies listed in the PEDro database. Br Med Bull.

[8] Yeaman LD, Jerome CP, McCullough DL (1989). Effects of shock waves on the structure and growth of the immature rat epiphysis. J Urol.

[9] Nassenstein K, Nassenstein I, Schleberger R (2005). Effects of high-energy shock waves on the structure of the immature epiphysis–a histomorphological study. Z Orthop Ihre Grenzgeb.

[10] Van Arsdalen KN, Kurzweil S, Smith J, Levin RM (1991). Effect of lithotripsy on immature rabbit bone and kidney development. J Urol.

[11] Hamisultane R, Gordon R, Russo S, Kuderna H, Auersperg V, Schaden W, Thiele RCR. Consensus statement recommendations for the use of extracorporeal shockwave technology in medical indications. ISMST Annu Gen Meet Juan les Pins; 2008. p. 3–5

[12] Lohrer H, Nauck T, Schöll J, Zwerver J, Malliaropoulos N (2012). Extracorporeal shock wave therapy for patients suffering from recalcitrant osgood-schlatter disease. Sportverletzung-Sportschaden.

[13] Lohrer H, Nauck T (2015). Radiale extrakorporale Stoßwellentherapieb zur Behandlung der Apophysitis calcanei. Dtsch Z Sportmed.

[14] Eid J. Consensus statement on ESWT indications and contraindications. WwwShockwavetherapyOrg; 2016. p. 2–4

[15] Shanmugam C, Maffulli N (2008). Sports injuries in children. Br Med Bull.

[16] Longo UG, Ciuffreda M, Locher J, Maffulli N, Denaro V (2016). Apophyseal injuries in children’s and youth sports. Br Med Bull.

[17] Maffulli N, Baxter-Jones AD (1995). Common skeletal injuries in young athletes. Sports Med.

[18] Loske AM (2017). Shock waves as used in biomedical applications. Medical and biomedical applications of shock waves.

[19] Amer MA (2021). Letter regarding: effectiveness of extracorporeal shockwave therapy in the treatment of chronic insertional Achilles tendinopathy. Foot Ankle Int.

[20] Arnold A, Thigpen CA, Beattie PF, Kissenberth MJ, Shanley E (2017). Overuse physeal injuries in youth athletes: risk factors, prevention, and treatment strategies. Sports Health.

[21] Ryskalin L, Morucci G, Natale G, Soldani P, Gesi M (2022). Molecular mechanisms underlying the pain-relieving effects of extracorporeal shock wave therapy: a focus on fascia nociceptors. Life.

[22] Simplicio CL, Purita J, Murrell W, Santos GS, dos Santos RG, Lana JFSD (2020). Extracorporeal shock wave therapy mechanisms in musculoskeletal regenerative medicine. J Clin Orthop Trauma.

[23] d’Agostino MC, Craig K, Tibalt E, Respizzi S (2015). Shock wave as biological therapeutic tool: from mechanical stimulation to recovery and healing, through mechanotransduction. Int J Surg.

[24] Abe Y, Ito K, Hao K, Shindo T, Ogata T, Kagaya Y, Kurosawa R, Nishimiya K, Satoh K, Miyata S, Kawakami K, Shimokawa H (2014). Extracorporeal low-energy shock-wave therapy exerts anti-inflammatory effects in a rat model of acute myocardial infarction. Circ J.

[25] Lana JF, Macedo A, Ingrao ILG, Huber SC, Santos GS, Santana MHA (2019). Leukocyte-rich PRP for knee osteoarthritis: current concepts. J Clin Orthop trauma.

